# Cancer-associated fibroblast-derived gene signatures determine prognosis in colon cancer patients

**DOI:** 10.1186/s12943-021-01367-x

**Published:** 2021-04-29

**Authors:** Mercedes Herrera, Alberto Berral-González, Igor López-Cade, Cristina Galindo-Pumariño, Santiago Bueno-Fortes, Manuel Martín-Merino, Alfredo Carrato, Alberto Ocaña, Carolina De La Pinta, Ana López-Alfonso, Cristina Peña, Vanesa García-Barberán, Javier De Las Rivas

**Affiliations:** 1 Department of Oncology-Pathology, Karolinska Institutet, Stockholm, Sweden; 2 Bioinformatics and Functional Genomics Group, Cancer Research Center (CiC-IBMCC, CSIC/USAL), Consejo Superior de Investigaciones Científicas (CSIC) and University of Salamanca (USAL), Salamanca, Spain; 3 Molecular Oncology Laboratory, Instituto de Investigación Sanitaria San Carlos (IdISSC), Madrid, Spain; 4 Medical Oncology Department, Ramón y Cajal University Hospital, IRYCIS, CIBERONC, Alcalá University, Madrid, Spain; 5 Facultad de Informática, Universidad Pontificia de Salamanca (UPSA), Salamanca, Spain; 6 Experimental Therapeutics Unit, Instituto de Investigación Sanitaria San Carlos (IdISSC) and CIBERONC, Madrid, Spain; 7 Radio-Oncology Department, Ramón y Cajal University Hospital, IRYCIS, Alcalá University, Madrid, Spain; 8 Medical Oncology Department, Infanta Leonor Hospital, Madrid, Spain

**Keywords:** Colon Cancer, Tumor microenvironment, Cancer-associated fibroblasts, Exosomes, Noncoding RNAs, Prognostic signatures, Liquid biopsy

Paracrine communication between tumor and surrounding stroma arbitrates the malignant behavior of cancer progression [1]. Fibroblasts, which are the main cell type within the stroma and are called cancer-associated fibroblasts (CAFs), orchestrate the crosstalk with cancer cells [2, 3] and express several markers associated with prognosis [4]. There is increasing evidence that a stroma-specific signature could be used for risk assessment in colon cancer (CC). According to the Consensus Molecular Subtype classification (CMS) in CC, the mesenchymal or CMS4 group is characterized by stromal invasion, extracellular matrix remodeling and TGF-β signaling activation. It is associated with the worst prognosis rates [5, 6]. Genes correlating with the mesenchymal subtype are mostly expressed by CAFs and other stromal cells, rather than by tumor cells [7]. Accordingly, our group defined a gene expression profile associated with CAFs with high pro-migratory effects on colon tumor cells, which was associated with patients’ poor prognosis. These were mostly advanced-stage patients [8].

The crosstalk between tumor and stromal cells is conducted in part by exosomes that are involved in many tumorogenic processes. ncRNAs contained in exosomes secreted by colon CAFs enhance proliferation and stemness properties of tumoral cells and are involved in chemoresistance [9]. Recently, our group demonstrated that there was a difference in how CAFs and normal colon mucosa fibroblasts (NFs) distributed ncRNA into the exosomal cargo. There was the same difference in distribution for their potential ncRNA target genes in recipient cells [10].

Our research into CAF profiles and targets of CAF-derived exosome cargo in recipient cells found novel CAF-derived signatures with prognostic value in colon cancer patients. This showed the importance of CAFs and their derived exosomes in tumor progression (Fig. 1).

**Fig. 1:**
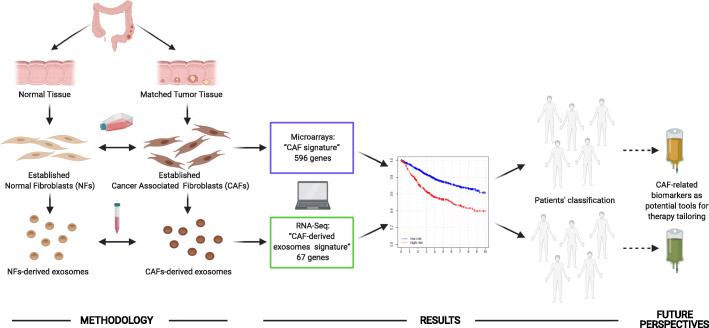
Diagram of the study and future perspectives. Workflow chart showing the process for identifying CAF-related signatures: “CAF signature” and “CAF-derived exosomes signature” with 596 and 67 genes, respectively. Patient classification is based on tumor microenvironment differences defined by CAF-derived signatures, in order to identify those patients with high/low risk of poor outcome. The results identify CAF-related biomarkers as potential tools for therapy tailoring to improve colon cancer patients’ survival. Created with BioRender.com

## CAF versus normal fibroblasts’ gene signature as a marker of risk and survival in colon cancer patients

**Fig. 2:**
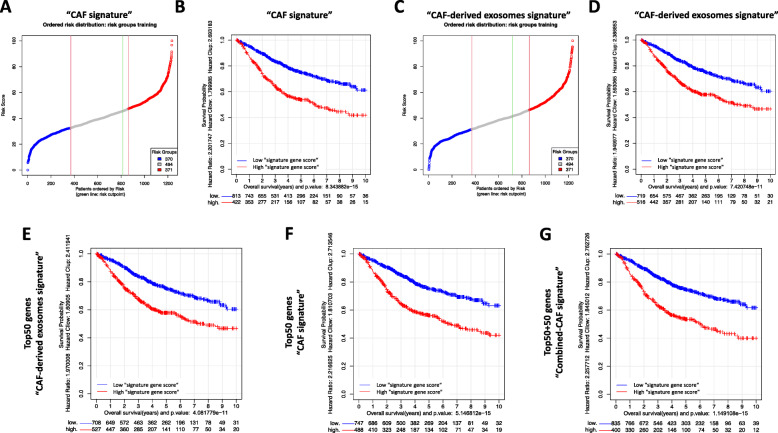
Risk and multivariate survival analyses of the colon cancer cohort (1235 tumor samples), divided in line with the CAF-related signatures. “CAF signature” (596 genes) showed prognosis value in colon cancer patients: **a** Distribution of risk scores from low (blue) to high (red). Risk scores were scaled from 1 to 100. The dots in gray in the middle correspond to intermediate scores; and the green line marks the point for the optimal split of samples into low- and high-risk ones; **b** Kaplan-Meier graph showing that the high-risk subset of patients had significantly shorter overall survival (OS) than the low-risk one (HR = 2.20; *p*-value = 8.34 E-15). “CAF-derived exosomes signature” (67 genes) showed prognosis value in colon cancer patients: **c** Distribution of risk scores from low to high; **d** Kaplan-Meier graph showing that the high-risk subset of patients had significantly shorter OS than the low-risk one (HR = 1.95; *p*-value = 7.42 E-11). Survival analysis using the top 50 genes of CAF-related signatures also found high- and low-risk patients: **e** Kaplan-Meier graph obtained using the top 50 genes of the “CAF-derived exosomes signature” (HR = 1.97; *p*-value = 4.08 E-11); **f** Kaplan-Meier graph with the top 50 genes of the “CAF signature” (HR = 2.22; *p*-value = 5.15 E-15); **g** Kaplan-Meier graph with the top 100 genes from the combination of both top 50s used in E and F (HR = 2.26; *p*-value = 1.15 E-15). In each case, the graph includes the survival traces of the patients with low risk (blue) versus the patients with high risk (red)

A “CAF signature”, involving 596 protein-coding genes, was identified when gene expression data from colon tumor CAFs and NFs were compared (Additional files 1 and 2). Functional gene-set analysis identified catabolic process, intracellular transport and dimerization and binding of proteins, among others, as enriched biological terms (Additional file 3). The prognostic value and clinical relevance of the new “CAF signature” were evaluated in a meta-dataset of 1235 colon cancer patients. Figure 2a shows the results of the risk prediction for each of the 1235 colon tumors and the best cut-off to divide patients into lower/higher “signature gene score”, based on expression levels of selected genes. Interestingly, the survival analysis showed that a high “signature gene score” subset of patients had shorter overall survival (OS) than the low “signature gene score” patients (Fig. 2b). In line with these data, the prognostic value of our previously reported “pro-migratory CAF signature” [8] was also confirmed in this cohort of 1235 patients (data not shown).

## Prognostic value of a novel CAF-derived exosomes signature

The ncRNA consists of functional RNA molecules that act by means of multiple mechanisms in the regulation of diverse cell functions [11]. In this study, we analyzed a second signature, “CAF-derived exosomes signature”, containing 67 predicted target genes of over-distributed ncRNA genes in CAF-derived exosomes previously described by our group [10] (Additional file 4). Gene set functional enrichment analysis identified important pathways related to cancer progression and microenvironment regulation, such as proliferation, positive regulation of signaling, regulation of multicellular organismal process and DNA repair processes, among others (Additional file 5). The risk cut-off point (Fig. 2c) of the “CAF-derived exosomes signature” in colon cancer was also evaluated in the same large meta-dataset (*N* = 1235), confirming the signature’s prognostic value (Fig. 2d).

## Overlap of CAF-related signatures and prognostic value, using their combination

Our first signature is related to CAF activation or status, while the second is associated with the crosstalk between fibroblasts and stromal/cancer cells orchestrated by CAF-derived exosomes. Both CAF-related signatures clearly overlapped in the classification of patients as at high or low risk of death (82.6% of concordance). To achieve a better and more balanced comparison of the behavior of the two CAF-related signatures, we undertook a new analysis of only the top 50 genes of each signature (Additional file 6), which confirmed their prognostic value (Fig. 2e and f). The combination of the two signatures (50 + 50 genes) did not greatly improve the separation of the survival curves of low- and high-“signature gene score” patients (Fig. 2g), indicating that there was no overlapping gene between the two gene signatures. Therefore, they include features that are closely related or measure similar characteristics in the colon tumors, though from two perspectives: CAF gene deregulation and fibroblasts/other cells’ crosstalk.

## Prognostic value of CAF-related signatures increases in advanced-stage tumors and in CMS4 tumors

**Fig. 3:**
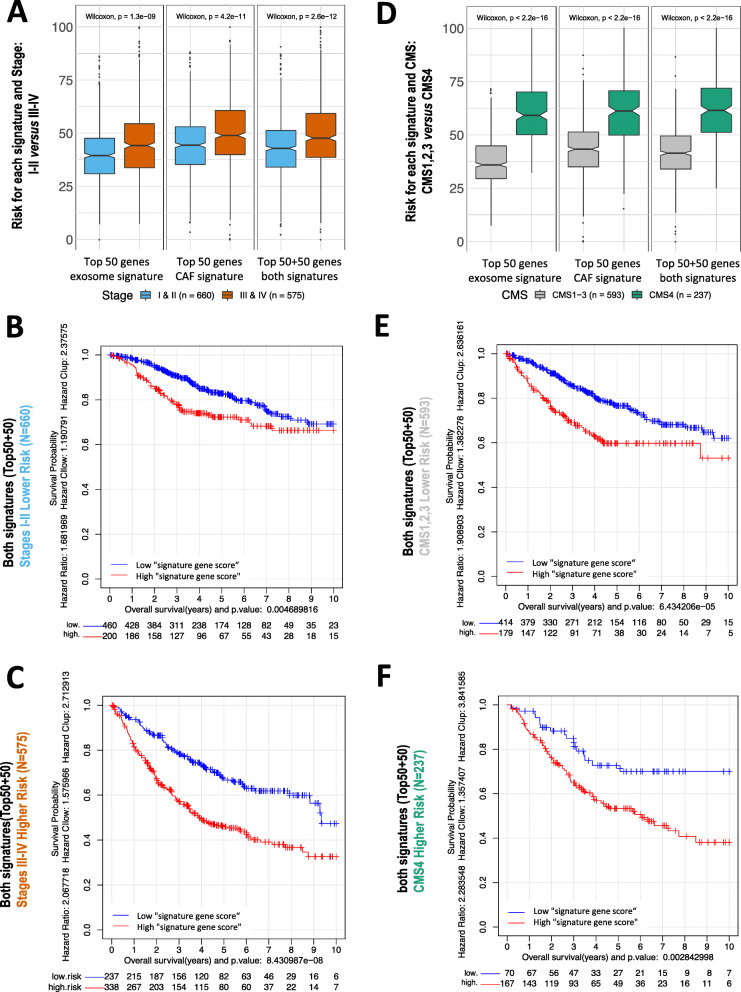
CAF-related signatures showed stronger poor-prognosis association in those patients with advanced colon tumors than in initial stages: Comparison of the prognosis of the colon tumors (1235 samples) in initial stages I and II (660 samples) and in advanced stages III and IV (575 samples). The risk assigned to each subgroup using 3 different gene signatures is shown in (**a**). The survival analysis examined the two sub-groups of samples, in stages I & II (**b**) or in stages III & IV (**c**), using the combination of both top 50 + 50 gene signatures. CAF-related signatures showed stronger poor-prognosis association in those patients with CMS4 than in CMS1–3. Comparison of the prognosis of the colon tumors classified following the consensus molecular subtypes (CMS1,2,3,4) defined by Guinney et al. [5, 11], divided into two groups: 593 samples of subtypes CMS1,2,3 and 237 samples of subtype CMS4. **d** Risk assigned to each of these 2 groups using the top 50 genes of signature 67, the top 50 of signature 596 and the top 50 + 50 from the two combined. Survival analysis using the combined signature for the 2 groups of samples separated: CMS1,2,3 (**e**) and CMS4 (**f**)

Initial analysis of survival of our cohort of patients confirmed the prognostic value of tumor stage (Additional file 7A). Interestingly, when we calculated the risk of each individual by splitting the population into stages (Fig. 3a), we found that all CAF-related signatures predicted higher risk at advanced stages than at early stages. In parallel, the prognostic value of CAF signatures was stronger in the stage III-IV group of patients than in the group of patients at early tumor stages (Fig. 3b-c and Additional file 8A). Our previous studies corroborated these data and showed the greater impact of microenvironment activation in patients’ outcome at advanced CC stages [8, 12]. Since these patients are usually those with worse outcomes, new therapies targeting microenvironment components would improve their clinical management. Specifically, stage III colon cancer patients are likely to develop recurrence. However, a subgroup of stage III patients would have low risk of recurrence and better outcome, with the advantage of shorter chemotherapy treatments that would avoid oxaliplatin cumulative neurotoxicity [13]. However, in daily clinical practice there is not enough evidence for better risk stratification in high/low-risk stage III patients. Our defined CAF-derived signatures with a clear prognostic value might improve patients’ classification and thus cancer patients’ management.

In addition, we confirmed that CMS4 patients had lower survival than CMS1, 2 or 3 ones (Additional file 7B). The Wilcoxon test revealed a higher “signature gene score” predicted by any of the 3 CAF-related signatures in CMS4-type tumors than in CMS1–3 ones (Fig. 3d). Although the Kaplan-Meier survival analysis showed that our CAF-related signatures marked differences in both CMS1–3 and CMS4 tumors (Fig. 3e-f and Additional file 8B), the comparison of the “signature gene score” distributions clearly indicated that in CMS4 risks were higher. CMS4-like subtypes and treatment associations have been studied in some clinical and preclinical studies. However, the clinical translation of the CMS subtypes into treatment decision-making is not widespread [14]. As our defined CAF-derived signatures had a higher prognostic value in those patients in the CMS4-like subtype, this might provide a new framework for patient classification in clinical trials and for the adjustment of suitable treatments. Therapies with the tumor microenvironment as the main target are currently an area of intensive research [15].

## Conclusions

In summary, clinical decisions are based on the usual histological and clinical parameters that do not accurately predict the biological behavior of histologically equal tumors. The risk of recurrence of CC cancer is a critical factor in correctly setting up therapeutic guidelines, which highlights the importance of studying prognosis and predictive biomarkers. These new biomarkers classify patients on the basis of tumor microenvironment differences and define those pathological events at the molecular and cell level associated with patients’ survival. Our results emphasize ongoing efforts to decipher CAF biomarkers in order to advance prognosis and chemotherapeutic responses’ heterogeneity and to improve patients’ survival by tailoring therapies based on specific biological characteristics of CC tumors.

## Supplementary Information

**Additional file 1.** Material and Methods; Clinical and phenotypic characteristics of the colorectal cancer cohort of 1273 patients analyzed in this study.

**Additional file 2 **List of 596 protein-coding genes defined by the “CAF signature”, including: gene symbol; gene description; ENSEMBL gene id; and statistical parameters provided by the SAM algorithm (i.e. fold change in log2 scale, statistic parameter d value, and *p* value) in the analysis of differential expression.

**Additional file 3.** Functional enrichment analysis with GO-BP and GO-MF of the genes of the “CAF signature” within the database MSigDB. The table shows the most relevant GO terms (top 20 in GO-BP and in GO-MF) associated with genes from the CAF-derived signature.

**Additional file 4.** List of the 67 genes included in the “CAF-derived exosomes signature”. For each gene the table gives the gene symbol and the ENSEMBL gene id (ENSG).

**Additional file 5.** Functional enrichment analysis with GO-BP and GO-MF of the genes of the “CAF-derived exosomes signature” within the database MSigDB. The table shows the most relevant GO terms (top 20 in GO-BP and in GO-MF) associated with genes from this signature.

**Additional file 6.** Top 50 genes of each signature (CAF-signature in blue and CAF-derived exosomes signature in green) and the beta-values corresponding to each gene provided by Cox. * non-coding genes.

**Additional file 7.** Survival analysis of colon cancer cohort including phenotypic factors: tumor stage and CMS. (A) Distribution of the patients at stages I & II versus III & IV clearly showed different prognoses. (B) CMS4 patients showed shorter survival than CMS1, CMS2 and CMS3 ones.

**Additional file 8.** CAF-related signatures showed stronger poor prognosis association in those patients with advanced colon tumors than in initial stages and in those patients with CMS4 than in CMS1–3. Comparison of the prognosis of the colon tumors (1235 samples) in initial stages I and II (660 samples) and in advanced stages III and IV (575 samples) (A), divided into CMS1–3 (593 samples) and CMS4 (237 samples) subtypes (B). The survival analysis used 2 gene signatures: the top 50 genes of the 67-gene signature (“CAF-derived exosomes signature”) and the top 50 of the 596-gene signature (“CAF signature”).

## Data Availability

All the data corresponding to the colorectal cancer series used in this study are available in GEO (https://www.ncbi.nlm.nih.gov/geo), which is a public functional genomics data repository. The source identifier (GEO GSM id) of each sample used is indicated in the corresponding Additional File.

## Abbreviations

CAF: Cancer-Associated Fibroblast
CC: Colon Cancer
CMS: Consensus Molecular Subtypes
CRC: ColoRectal Cancer
GO-BP: Gene Ontology-Biological Process
GO-MF: Gene Ontology-Molecular Function
HR: Hazard Ratio
ncRNA: Non-Coding RNA
NF: Normal Fibroblast
NGS: Next Generation Sequencing
