# Supplementary material for: Cancer-associated fibroblast-derived gene signatures determine prognosis in colon cancer patients
Source: Mol Cancer. 2021 Apr 29;20:73. doi: 10.1186/s12943-021-01367-x (PMC8082938; doi:10.1186/s12943-021-01367-x)
Supplement: Supplementary file 1 — Additional file 1. Material and Methods; Clinical and phenotypic characteristics of the colorectal cancer cohort of 1273 patients analyzed in this study. [file 12943_2021_1367_MOESM1_ESM.docx]

**Cancer-Associated Fibroblast-derived gene signatures determine prognosis in colon cancer patients**

**MATERIALS AND METHODS**

**Differential expression between primary colon CAFs and normal fibroblasts**

We used genome-wide expression data samples from CAFs and NFs described in our previous studies [16] (accession number GSE51257). These data were obtained from primary CAFs and NFs propagated from fresh primary tumors and normal tissue, respectively, of colon cancer patients. Gene expression was analyzed using the high-density microarrays from *Affymetrix*: GeneChip arrays (Human Gene 1.0 ST). For the current study we compared CAFs (4 samples, GSM1241296, GSM1241297, GSM1241298 and GSM1241299) and NFs (2 samples, GSM1241300, GSM1241301). The raw expression signal from the microarrays was normalized. Then, the differential expression was calculated, using the RMA algorithm [17] and assigning the signal values, not to *Affymetrix* probesets, but to ENSEMBL genes (as described in [18]); using the custom CDF *hugene10sthsengcdf* obtained from <http://brainarray.mbni.med.umich.edu/Brainarray/Database/CustomCDF/> [19]. For the differential expression we applied two alternative algorithms, SAM [20] and LIMMA [21], in both cases using unequal variances and selecting the genes with a significant p-value ≤ 0.05. The result using SAM provided a set of 622 genes and the LIMMA result, 972. Since we were comparing 6 samples to ensure a robust result, we selected the genes corresponding to the intersection of both sets that were significant in both analyses, which provided a final list of 596 protein-coding genes. All these analyses were carried out using R programming language for statistical computing (<https://www.r-project.org/>). Gene-annotation enrichment was analyzed to identify enriched biological themes (GO terms) for the selected signatures, using the “Molecular Signatures Database” (MSigDB) v7.2 [22,23].

**Colon cancer unified cohort including expression and survival data**

We prepared and analyzed the data from a large integrated cohort of colon primary tumors (N=1,273 samples) [24], which included 7 data series of colorectal cancer with genome-wide expression data plus survival data and other clinical information of the patients. The set of 38 rectal tumors included in our initial colorectal cancer cohort were not included in these survival and risk analyses, in order to focus our study on colon adenocarcinomas (N=1,235 colon cancer samples). All the colon cancer samples included in this dataset were tested for global gene expression profiling by the platform of high-density microarrays from *Affymetrix*: Human Genome U133 Plus 2.0. As indicated above, the dataset also contained phenotypical and clinical information about the patients, i.e., age at diagnosis, gender, survival time, cancer stage and tumor location. The samples corresponded in all cases to primary tumors without pre-operative chemotherapy and/or radiotherapy. Phenotypical data of the cohort studied (when information was not available for a given sample, the data table included *not assigned* values (NA)):

Using the genome-wide expression data from the colon cancer samples of our integrated dataset, we applied two predictive algorithms to assign the Consensus Molecular Subtypes (CMS) classification defined by [25] to each tumor sample of our cohort. With the two algorithms used for this classification, *CMSclassifier* [25] and *CMScaller* [26], we obtained an accurate significant assignment of 830 colon samples to CMS (in 4 categories: CMS1, 2, 3 or 4).

**Colon cancer cohort risk prediction and survival analysis with different gene signatures**

To evaluate the prognostic value of different gene signatures in a cohort of colon cancer tumors, a robust version of the multivariate Cox regression model was applied. In particular, we used regularized multivariate Cox proportional-hazards regression with L_1_ norm penalty [27], with the scope to build a multigenic risk predictor. A recursive algorithm using double-nested cross-validation with optimization of regression parameters searched for the value-of-risk score that best split the cohort into two groups: low risk and high risk. Moreover, the penalty procedure shrunk to zero the coefficient of any feature of the multivariate model (i.e. any gene) not used to predict the risk. Thus, it selected the features that had more power when the prediction was computed, providing a score (that can be interpreted as a ranked coefficient) for each feature used. Once each patient’s risk had been calculated, a Kaplan-Meier analysis checked the separation of the two groups according to the survival data: (i) a high-risk group of individuals (with poor survival, plotted in red) and (ii) a low-risk group of individuals (with good survival, plotted in blue). A log-rank test evaluated the difference between the Kaplan-Meier curves of the two groups of patients for each gene signature tested. This statistical test is non-parametric and makes no explicit assumptions about the form of the survival curves. The genes, sets of genes or gene signatures that split the patients into groups with different prognoses that had a statistically significant p-value were considered "survival markers": the best markers were the features (i.e. the genes) that showed the largest beta-values inside the multivariate model. All these procedures and methods were developed and applied using R (https://www.r-project.org/) programming language for statistical computing.

All these analyses were carried out in the cohort of 1,235 samples from patients with colon cancer, using for multivariate analysis the genes included in the CAF signature (596 genes) or the genes included in the signature of CAF-derived exosomes (67 genes). In addition, the top 50 genes of each of these signatures were tested in the same way. These genes were selected by using the beta-values that the multivariate Cox regression provided for each gene when using the whole signatures. In the exploratory sub-set analyses defined by tumor stage and the consensus molecular subtypes, initial analysis of survival of our cohort of patients in relation to these characteristics is showed in Additional File 7. It should be noted that the risk value annotated by CAF-derived signatures is statistically different for tumor stage and CMS classification.

**References:**

16. Herrera M, Islam ABMMK, Herrera A, Martín P, García V, Silva J, et al. Functional heterogeneity of cancer-associated fibroblasts from human colon tumors shows specific prognostic gene expression signature. Clin Cancer Res. 2013;19:5914–26.

17. Irizarry RA, Hobbs B, Collin F, Beazer-Barclay YD, Antonellis KJ, Scherf U, et al. Exploration, normalization, and summaries of high density oligonucleotide array probe level data. Biostatistics. 2003;4:249–64.

18. Risueño A, Fontanillo C, Dinger ME, De Las Rivas J. GATExplorer: Genomic and Transcriptomic Explorer; mapping expression probes to gene loci, transcripts, exons and ncRNAs. BMC Bioinformatics. 2010;11:221.

19. Dai M, Wang P, Boyd AD, Kostov G, Athey B, Jones EG, et al. Evolving gene/transcript definitions significantly alter the interpretation of GeneChip data. Nucleic Acids Res. 2005;33:e175.

20. Tusher VG, Tibshirani R, Chu G. Significance analysis of microarrays applied to the ionizing radiation response. Proc Natl Acad Sci U S A. 2001;98:5116–21.

21. Ritchie ME, Phipson B, Wu D, Hu Y, Law CW, Shi W, et al. Limma powers differential expression analyses for RNA-sequencing and microarray studies. Nucleic Acids Res. 2015;43:e47.

22. Subramanian A, Tamayo P, Mootha VK, Mukherjee S, Ebert BL, Gillette MA, et al. Gene set enrichment analysis: A knowledge-based approach for interpreting genome-wide expression profiles. Proc Natl Acad Sci U S A. 2005;102:15545–50.

23. Liberzon A, Subramanian A, Pinchback R, Thorvaldsdóttir H, Tamayo P, Mesirov JP. Molecular signatures database (MSigDB) 3.0. Bioinformatics. 2011;27:1739–40.

24. Martinez-Romero J, Bueno-Fortes S, Martín-Merino M, Ramirez De Molina A, De Las Rivas J. Survival marker genes of colorectal cancer derived from consistent transcriptomic profiling. BMC Genomics. 2018;19(Suppl8):857.

25. Guinney J, Dienstmann R, Wang X, De Reyniès A, Schlicker A, Soneson C, et al. The consensus molecular subtypes of colorectal cancer. Nat Med. 2015;21:1350–6.

26. Eide PW, Bruun J, Lothe RA, Sveen A. CMScaller: An R package for consensus molecular subtyping of colorectal cancer pre-clinical models. Sci Rep. 2017;7:16618.

27. Gui J, Li H. Penalized Cox regression analysis in the high-dimensional and low-sample size settings, with applications to microarray gene expression data. Bioinformatics. 2005;21:3001–8.
