# Supplementary figures and images for: Cancer-associated fibroblast-derived gene signatures determine prognosis in colon cancer patients
Source: Mol Cancer. 2021 Apr 29;20:73. doi: 10.1186/s12943-021-01367-x (PMC8082938; doi:10.1186/s12943-021-01367-x)

Additional File 7

A

Stages I-II & III-IV (1235 samples)

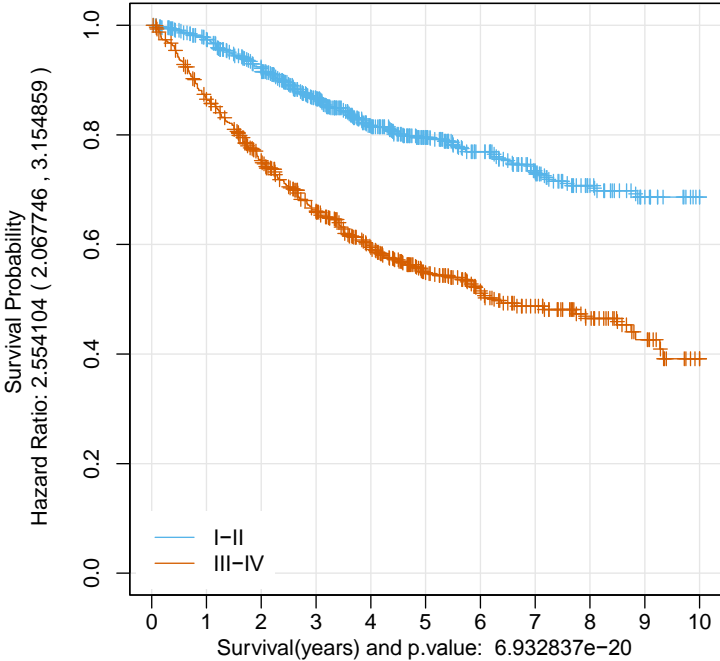

|        |     |     |     |     |     |     |     |     |    |    |    |
|--------|-----|-----|-----|-----|-----|-----|-----|-----|----|----|----|
| I-II   | 660 | 614 | 542 | 438 | 334 | 241 | 183 | 125 | 77 | 53 | 38 |
| III-IV | 575 | 482 | 390 | 310 | 235 | 162 | 123 | 83  | 51 | 30 | 13 |

B

CMS1,2,3 & CMS4 (830 samples)

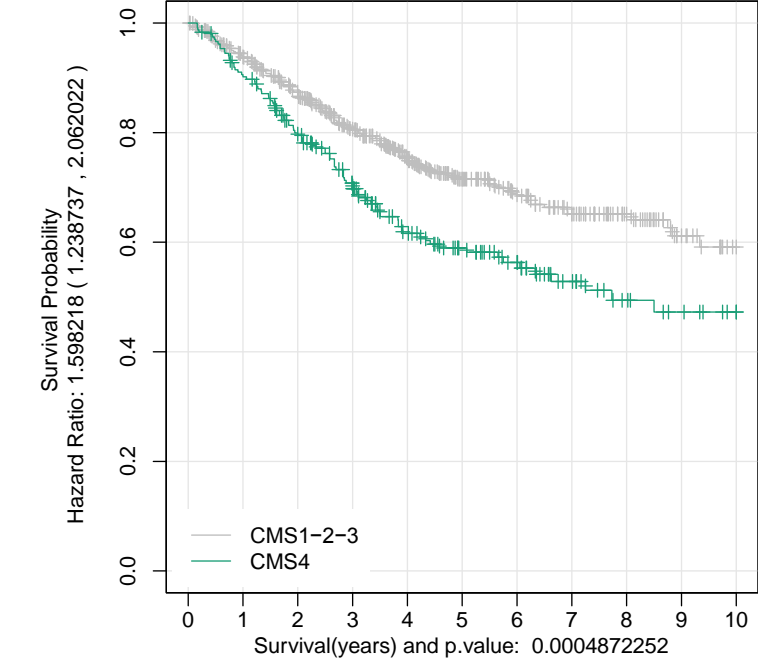

|          |     |     |     |     |     |     |     |     |    |    |    |
|----------|-----|-----|-----|-----|-----|-----|-----|-----|----|----|----|
| CMS1-2-3 | 593 | 526 | 452 | 362 | 283 | 192 | 146 | 104 | 64 | 36 | 20 |
| CMS4     | 237 | 210 | 175 | 140 | 98  | 76  | 57  | 38  | 25 | 19 | 13 |

Supplement: Supplementary file 7 — Additional file 7. Survival analysis of colon cancer cohort including phenotypic factors: tumor stage and CMS. (A) Distribution of the patients at stages I & II versus III & IV clearly showed different prognoses. (B) CMS4 patients showed shorter survival than CMS1, CMS2 and CMS3 ones. [file 12943_2021_1367_MOESM7_ESM.pdf]

Additional File 8

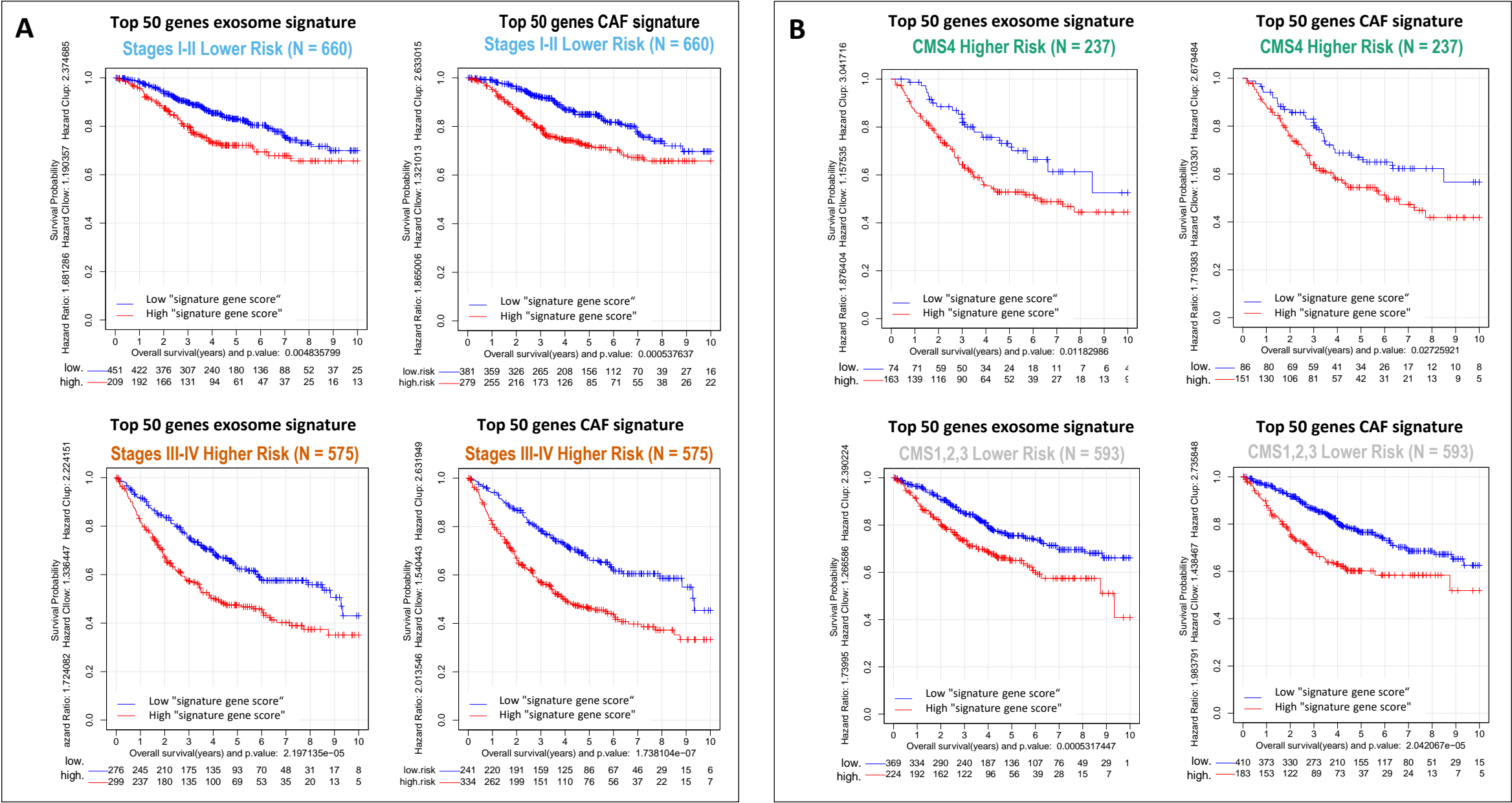

Supplement: Supplementary file 8 — Additional file 8. CAF-related signatures showed stronger poor prognosis association in those patients with advanced colon tumors than in initial stages and in those patients with CMS4 than in CMS1–3. Comparison of the prognosis of the colon tumors (1235 samples) in initial stages I and II (660 samples) and in advanced stages III and IV (575 samples) (A), divided into CMS1–3 (593 samples) and CMS4 (237 samples) subtypes (B). The survival analysis used 2 gene signatures: the top 50 genes of the 67-gene signature (“CAF-derived exosomes signature”) and the top 50 of the 596-gene signature (“CAF signature”). [file 12943_2021_1367_MOESM8_ESM.pdf]
